# Toward a comprehensive taxonomy of human motives

**DOI:** 10.1371/journal.pone.0172279

**Published:** 2017-02-23

**Authors:** Jennifer R. Talevich, Stephen J. Read, David A. Walsh, Ravi Iyer, Gurveen Chopra

**Affiliations:** 1 Department of Psychology, University of Southern California, Los Angeles, CA, United States of America; 2 Ranker, Los Angeles, CA, United States of America; University of Würzburg, GERMANY

## Abstract

A major success in personality has been the development of a consensual structure of traits. However, much less progress has been made on the structure of an equally important aspect of human psychology: motives. We present an empirically and theoretically structured hierarchical taxonomy of 161 motives gleaned from a literature review from McDougall to the present and based on the cluster analysis of similarity judgments among these 161 motives, a broader sampling of motives than previous work. At the broadest level were: Meaning, Communion, and Agency. These divided into nine clusters: Morality & Virtue, Religion & Spirituality, Self-Actualization, Avoidance, Social Relating, Family, Health, Mastery & Competence, and Financial & Occupational Success. Each divided into more concrete clusters to form 5 levels. We discuss contributions to research on motives, especially recent work on goal systems, and the aiding of communication and systematization of research. Finally, we compare the taxonomy to other motive organizations.

## Introduction

Goals and motives are fundamental to human behavior: they play a central role in its enactment and in our understanding of why people do what they do. Moreover, they have long been considered essential aspects of human personality (e.g., [1–12]). Yet despite their essential role in human behavior, we have only an incomplete idea of how human motives are structured and organized [13]. Although a variety of different motive lists and small-scale taxonomies have been proposed, psychologists have not yet developed a comprehensive, empirically based structure of human motives.

This is unfortunate because a common conceptual framework systematizes and integrates knowledge; it greatly advances research and its application by increasing the field’s ability to understand, predict, and influence its object of study—in our case, human behavior. With a common frame of reference, communication among researchers is aided, and repetitive, overlapping efforts can be avoided. Moreover, a comprehensive structure provides a rich resource for research: it provides numerous hypotheses about the mechanisms underlying behavior and it provides a basis for measurement and comparison across individuals and studies. The result is that research is facilitated and the pace of theory development is accelerated.

### The advantages of a comprehensive structure

With the recent explosion of interest in human goal systems and their dynamics, researchers have argued that understanding the structure of human motives is central to understanding motivation (e.g., [14–16]. For example, Kruglanski et al.’s [16] Goal Systems Theory has argued that the cognitive structure of goals represents how goals are related to one another: which goals are pursued together, which conflict, and which are pursued independently. When a goal is activated, we may use the goal structure to predict which other goals are likely to be primed or inhibited.

Carver and Scheier’s [14] cybernetic model of the self-regulation of behavior (see also [15]) has noted that goals are often hierarchically organized, with higher order goals being broader and more abstract. This hierarchical organization has several important implications: 1) higher order goals are more likely to function as general principles guiding behavior, and related to this, 2) higher order goals are likely to play a directing or controlling role over a much wider range of behavior than are more concrete, lower order goals. In contrast, 3) lower level goals have much greater specificity and apply to a much more restricted set of behaviors.

We should note that researchers often use the term goals and motives interchangeably. However, there are important distinctions. Motives have force or “energy”, are things that people want. They provide the energizing or driving force behind behavior[17,18]. In contrast, goals do not *necessarily* have hedonic value or force. Frequently, they can simply be used to describe a step in a hierarchy of behavior. They are cognitive representation of an end state. For instance, in the Artificial Intelligence Planning literatures or in Carver and Scheier’s [14] cybernetic model, behavioral sequences are organized in goal-subgoal hierarchies, where a goal can simply be part of carrying out a sequence. Walking across the room or picking up a glass may be goals, but in and of themselves they have little if any hedonic or affective value. But wanting water when one is thirsty or wanting to be with a friend when one is lonely does have energizing force. In the current work we use the term motive because we are focused on developing a taxonomy of things that have energizing or driving force. What researchers call goals sometimes have these characteristics, but frequently they do not.

A comprehensive taxonomy of human motives would provide us with information about these and other central aspects of motive systems. Taxonomies such as the periodic table of the elements and classifications of the biological world have played a major role in theory development in other sciences. Such taxonomies bring order to a body of knowledge and often help reveal important underlying principles in a domain. In so doing, they enhance a field’s capacity to understand, account for, and manipulate their complex subject matter. As John [19] noted in his discussion of the Big Five and its role in the field of personality, development of an adequate taxonomy of a scientific domain often plays a number of fundamental roles in the development of a field. Thus, a comprehensive motive structure would greatly facilitate a wide range of work on the role of motives in human behavior.

Psychology underutilizes taxonomic classification—perhaps because the things we seek to organize are not physical or directly observable. But we can see the advantages of taxonomic categorization in work with the Big Five (e.g., [20,21]; for a recent review see [22]) and related structures of human personality (e.g., [23,24]). The Big 5 taxonomic structure is widely viewed as one of the major accomplishments of the field of personality. It provides personality researchers with a commonly accepted framework for organizing a wide range of personality measures and for research and thinking about the nature of human personality and its role in human behavior. A comprehensive taxonomy of human motives could arguably play an even more important role across all the multiple fields that study human behavior.

Our purpose is to build a comprehensive structure of human motives that will be useful to researchers, in nearly any domain, who have questions about what matters to their participants. It would be a powerful tool for identifying the “right variables” to measure for a wide range of investigations into human motivation and help researchers identify both what matters within a particular domain, as well as across a range of domains. With a common framework, we could compare results across individuals and across domains.

A non-taxonomic method often employed to study the role of goals and motives in behavior is to simply ask participants to generate their own goals (e.g., [4,7,25–27]). However, this does not allow for easy comparison of results across individuals, different studies, or domains. With a taxonomic reference for these efforts, researchers could move on to the meat of their work–be it the study of cross-cultural values or encouraging mental patients to take their medication [28].

Our aim is to provide the most comprehensive collection of motives yet drawn from the literature–from past efforts both empirical and theoretical (see Generating a List of Motives). From this collection we create a multi-level structure that moves smoothly from a set of specific, concrete motives up into highly abstract conceptual categories. This should allow the researcher to “zoom in” or “out” to assess human motivation at different levels of specificity or generality. We then examine our structure for convergence with theories about motives throughout history (in the Discussion see Comparison With Previous Categorizations of Human Motivators). We expect to find that our taxonomy includes the key elements of past works but, because of its more comprehensive start, we will arrive at a more comprehensive end.

### Existing taxonomies: Generating a list of motives

The development of a comprehensive list of human motives has been one focus of work on motivation (see [13]). However, as Ford and Nichols [29] have noted, little consensus exists concerning such a list. Over the years, a wide variety of different suggestions have been made. In the 1930s, McDougall [30] presented a list of 13 instincts and Murray[10] posited 44 “variables of personality” as forces determining behavior. Later, Maslow [8] introduced a hierarchy of 5 kinds of human needs; Cattell [31] presented 16 “Ergs;” Rokeach [32,33] generated a list of 18 instrumental (ends) and 18 terminal (means) values; and Schank and Abelson [34] proposed 6 motive types. Wicker et al. [35] generated a list of 56 motives. Schwartz [36,37] argued for 10 major value types, recently expanded to 19 [38]. Grouzet et al. [39] identified 57 different goals organized into 11 categories. Bugental[40] argues for five major clusters of human motivations, Fiske [17] argues for five core motives, Bernard and Lac [41] present a measure of 15 evolutionary based motives, and Kenrick et al. [42] argue for eight major clusters of human motivations. Ozer ([43]) has also recently argued for a hierarchical goal taxonomy with eight broad categories of goals at the highest level (What he calls goals, particularly at the highest level, are what we are referring to as motives). In the discussion, we will compare the specific motive clusters in our taxonomy with these theories.

The range of motives that have been proposed is wide. The variability in how they have been structured is high. What we lack is a clear consensus on the space of human motivation and how it is structured. In the current project we attempt to draw upon all the different major proposals and descriptions of individual motives that have been offered by using these earlier works as the starting point for building the content of our taxonomy

### Generating a taxonomy: Theoretical or empirical?

#### Lessons from the big five

The Big Five is a comprehensive hierarchical taxonomy of personality traits. Over and over again, the Big Five personality traits emerge in factor analyses. The existence of the Big Five is widely accepted, although their meaning and the processes underlying them are somewhat murky [44]. However, whether researchers are utilizing or criticizing it, the Big Five has been a vibrant source of research for decades.

Taking our lessons from the Big Five, psychology’s most successful taxonomy, we see the power of empirical derivation to provide a consistent and comprehensive tool upon which researchers may base future scales and measures. And yet, it is crucial that the resultant structure be supported by the theories of our field–for theory will be the explanation for what underlies this structure.

### Empirical and theoretical motive structures

Some approaches to the creation of a structure of human motives have been based on theoretical and conceptual analyses while others have taken a more empirical approach (for further discussion see [13]). Our approach here is to start with an empirical approach and then relate our results to theoretical proposals. This strategy aims to avoid a key problem: starting with theory for such an endeavor can strongly constrain what we look for, and because it constrains where we look, it can limit what we find.

Among the earliest approaches were those of Murray [10], McDougall [30], and Maslow [8], who argued for frameworks based on theory. This approach continues with more recent proposals by Bugental [40] and Kenrick et al. [42], who base their work on evolutionary theory, and Fiske’s [17] work based on personality and social psychological theory.

Others have taken an empirical approach, gathering a wide range of motives, asking participants to make judgments about the motives, such as their importance or their similarity, and then analyzing the judgments to try to uncover the underlying structure. For example, Wicker et al. [35] generated a list of 56 motives, asked subjects to rate the importance of each motive (“How much do you want it?”), and then analyzed the ratings using factor and cluster analysis. Unfortunately, this did not group together items that were conceptually similar. As we argue below, importance ratings may not be the best way to uncover motive structure.

A more recent empirical approach, Chulef, Read, and Walsh’s [45] taxonomy of human motives, relied on similarity judgments, rather than importance ratings. They had participants sort 135 motives into groups based on which went together. The sorting results were then cluster analyzed to create an empirically derived hierarchical taxonomy of human motives.

Other approaches mix the empirical and theoretical. For example, Braithewaite and Law [46] started with the items in Rokeach’s Values Survey [32,33] and then examined its structure. More recently, Grouzet et al. [39] developed a set of 57 goal items that measured 11 general goal categories, based on a review of the literature and theoretical considerations, and then had subjects across 15 cultures indicate how important each goal item was. They then examined the factor structure of their 11 goal categories and subsequently examined the structure of the 11 categories in terms of a circumplex. They identified two major dimensions: Intrinsic versus Extrinsic and Self-Transcendence versus Physical Self. Schwartz and Bilsky [36,37] constructed a theoretically motivated three-tiered structure of values. Working top-down from the highest-order theoretical value categories (biological needs, interpersonal interactions, and group-level societal demands) they proposed seven (later 10) more concrete domain categories (e.g. Enjoyment Domain). Working from their domain categories they then picked specific values that seemed to fit into each domain (e.g. “having a comfortable life,” from Rokeach [32]). Recently, Schwartz et al. [38] have theorized a lower, more fine-grained, level of 19 values. The result of Schwartz’s work was a circumplex, with congruent values adjacent and incongruent values on opposite sides of the wheel. This circumplex has been extensively tested in many different countries.

Although theory driven approaches have been quite useful, what we find is often constrained by the framework that guides our search. An empirically based structure that starts from a comprehensive search of possible human motives, rather than from theoretical concerns should largely avoid these constraints. Thus, the focus of the current project is to develop an empirically based hierarchical taxonomy of human motives. We will examine whether and how the resulting structure fits with various theories, but we will largely rely on data and not theory to structure our taxonomy.

#### Selecting a basis for structure: Importance vs. similarity

In addition to determining the list of motives, we also need to decide how to determine the structure of human motives. As noted above, previous empirical approaches to creating motive taxonomies have used different bases; some used importance ratings ([35–39]) and others used similarity or meaning based judgments (e.g., [45]). In the current project we use similarity or meaning-based judgments. Importance and similarity provide very different information about the nature and structure of motives, as Lewin’s[6,47] Force Field Theory of motivation and Kruglanski et al’s [16] recent goal systems theory make clear.

Kruglanski et al [16] have noted that goal(or motive) systems have two major properties: structure and allocation. Structural properties concern the cognitive-interconnectedness of goal systems: how different goals and motives are related to one another; which are closely related, and which may be completely opposed. In contrast, allocational properties have to do with how limited resources are distributed toward the attainment of different goals and motives. The importance of different goals tells us how an individual is likely to allocate limited resources to the pursuit of different goals. Similarity tells us about the conceptual structure of goals and motives, how they are related to one another. These are very different properties.

Lewin’s Force Field Theory[6,47], a highly influential theory of motivation, provides an additional perspective on the differences between motivational importance and motivational structure. Lewin characterized motivational force in terms of a vector, which has three properties: direction, point of application, and strength (for a quick review see [3]).

*Point of application* simply means what content the motivational force is being applied to–the goal content. This is also related to the cognitive structural property of Goal Systems[16], how goals are cognitively related to one another, as we discussed above. Our taxonomy would in part, represent content.

Lewin’s *strength* of a vector refers to the amount of tension (or “need”) and is a function of psychological distance (growing stronger, as distance decreases, in anticipation). It is the vigor with which one moves, not the direction of movement. It’s most familiarly operationalized as importance ratings [16]. Strength of wanting says nothing directly about the content or meaning of the motive, or about the routes and barriers between one’s self and motive attainment. From the perspective of Goal Systems Theory [16] the strength of a force is an allocational property: it is used to determine the allocation of resources.

The *direction* of a force is determined by where one is in relation to one’s motive and what obstacles lie between. Direction is often operationalized as likelihood of attainment or, in our work, congruence ratings (discussed in the next section).

Thus, Lewin’s Force Field theory and Kruglanski et al’s Goal Systems theory argue that importance judgments and similarity judgments should tap into different kinds of information, and as a consequence, should result in different structures. Similarity judgments would give a conceptual, meaning based structure and would provide information about how motives are cognitively or conceptually related. Allport [1] and others (for a review see [22]) argued that when examining the structure of traits, important regularities in human behavior are encoded in the language people use to talk about social interaction. A similar argument can be made for motives. In contrast, importance information provides a strength or tension-based diagram of how a force is allocated within the motivational system. While this is of utmost interest when examining cultural and individual differences in goal pursuit activities, this is not a motivational structure, as Kruglanski has defined it, of contents (the points of application of motivational force). Thus, the structure of motives, as measured by similarity judgments, and individual or cultural differences in how motivational force is allocated within said structure, are distinct and equally essential. And though there has been extensive work conducted with importance ratings, there has been hardly any that can speak to the conceptual structure of motive contents. The current work focuses on understanding the conceptual structure of motives, based on the content of the motives, and not on their allocational strength or importance. Thus, our work will focus on measuring the similarity between motives and not on their importance.

### The current approach

Having people generate their own categories would provide us with valuable information concerning the organization of the motivational structures that underlie and guide people’s behavior. This approach will help provide a common language to describe and categorize motivational constructs.

A properly constructed taxonomy will include varying levels from low to high degrees of resolution [13]: it is a multi-level structure that moves smoothly from a set of specific, concrete motives up to highly abstract conceptual categories. This allows the researcher to “zoom in” or “out” to assess human motivation at different levels of specificity or generality. This is a central aim of the current work.

Second, the sample of participants should be sizable and cover a broad range of ages and demographics. This increases the stability and generalizability of the cluster solutions upon which the taxonomy is based. This used to be difficult to accomplish for a similarity-sorting task. Previously, such a task involved one subject, a deck of 135 3x5 cards, and the only surface large enough to sort them on: the floor of an empty room. Thanks to technological advances, a similarity-sorting task of this magnitude can now be administered on a computer and online.

It is critical that the compiled list of motives be very broad. Frequently under-sampled in such lists are the things that people are motivated to avoid, such as social rejection or anxiety. A growing body of work clearly distinguishes between two motivational systems: an Approach system that governs approach to rewarding stimuli and an Avoidance system that governs avoidance of punishing or aversive stimuli (e.g., [48–50]). A comprehensive taxonomy needs to adequately cover both.

And finally, a taxonomy must be as useful as it is theoretically interesting. In the discussion, we will refer to evidence for the usefulness of the new taxonomy in predicting and understanding a range of important life decisions and behavior (e.g., retirement, voluntary employee turnover, adherence to taking psychotropic medication, weight management, and communication in close relationships).

We began this enterprise over a decade ago with an earlier effort. In Chulef, Read, and Walsh [45] we first generated a set of 135 motives, based on an extensive review of the literature on human motivation. This was a much larger list of human motives than had been previously examined. We then asked naïve subjects to sort the motives into categories on the basis of their semantic similarity and applied a hierarchical cluster analysis to their judgments. The result was a similarity-based, hierarchically organized list of motives that was the beginnings of a taxonomy.

But this early effort [45] lacked several features necessary for a comprehensive taxonomy. First, although hierarchical, it did not have an explicit multi-level structure. An ability to “zoom in” or out at explicit levels of abstraction or detail, would allow researchers to systematically measure motives at different levels. Second, it did not include a large or broad sample of subjects. But a comprehensive taxonomy should be able to generalize broadly. Third, it was almost exclusively composed of approach motives, to the exclusion of the avoidance motivational domains. And it missed motives in several important domains. Our empirical work on the conceptual structure of trait terms makes it clear that several important motivational domains (e.g., purity, being responsible and on time, communicating with others, being lazy, empathy and making others happy) were not represented in our original set of motives. Work on the conceptual structure of traits can be informative for the current endeavor as motives are central to the meaning of many traits and are often used to measure aspects of traits in standard trait measures. Further, some of the clusters were represented by only 2 motives, which did not provide for a stable cluster. Fourth, its usefulness in predicting important life decisions and behavior was not examined. However, it provided a strong starting point as we work toward a comprehensive taxonomy of human motives.

The current taxonomy addresses all of these issues that were not addressed in our previous work. This work aims to create an empirically-generated conceptual (semantic) hierarchy to identify multiple levels: from fine-grained and practical to the kind of abstract clusters that fascinate theorists. To do this, we start with a large number (161) of specific motives, allow our subjects to determine how they are categorized, and then we examine how these categories are hierarchically organized into ever-broader categories. Thus, our work can potentially provide more extensive information about the detailed organization of human motives. Our hope is that this hierarchy would support researchers in many aspects of their research, in a continuing effort to provide an empirical base from which to select motives relevant to their domain.

## Methods

### Motive selection

We started with the 135 motives in Chulef, Read, and Walsh [45], which were selected based on an extensive review of the motivational literature in psychology, as described in the introduction. We then added to that set of motives from several different sources. First, we drew very heavily from another of our previous web-based studies[51] that examined the conceptual components of trait terms (Motives often are a central component of a trait). 43 trait adjectives were taken from the 5 dimensions of Hofstee, de Raad and Goldberg's [52] Big 5 Circumplex model. Respondents were given descriptions of individuals with these 43 traits, and asked to list the motives that they thought would be held by such individuals. In coding the results we identified a number of motives that had not been included in Chulef, Read, and Walsh's set of 135 motives. In addition, the original set of motive terms in Chulef, Read, and Walsh included relatively few motives concerning things that people would try to actively avoid, such as social rejection or anxiety. Finally, we also drew on other recent theoretical accounts of motivation to add motives that are deemed central to those theories, but which were not represented in our original set of motives. One such example is “Avoiding impure acts”, which is related to the emotion of Disgust and to various aspects of religion. We ended up with 161 motives (Table 1).

**Table 1:** Abbreviations for Each Motive and the Full Text for Each Motive.

Abbreviation	Full Text
Peace	A world at peace.
DiffThings	Accomplishing difficult things, overcoming challenges.
Harmony	Achieving harmony and oneness (with self and the universe)
FinanSec	Achieving lifetime financial security.
PersGrwth	Achieving personal growth.
Salvation	Achieving salvation.
FineDesign	Appreciating fine design.
AvAnx	Avoiding anxiety.
AvCrit	Avoiding criticisms from others.
AvFail	Avoiding failure.
AvGuilt	Avoiding feelings of guilt.
AvImpure	Avoiding impure acts.
AvPhysHrm	Avoiding physical harm.
AvRegrets	Avoiding regrets.
AvReject	Avoiding rejection by others.
AvStress	Avoiding stress.
MoreAssert	Be less shy or more assertive.
BeatCompete	Beat people in a competition.
GoodParent	Being a good parent (teaching, transmitting values).
Leader	Being a leader, being in charge.
AnalyzeInfo	Being able to analyze and synthesize information.
AttractSexPart	Being able to attract a sexual partner.
MeetFinanNeeds	Being able to meet my financial needs.
TakeRisks	Being able to take risks.
Ambitious	Being ambitious, hard-working.
Ethical	Being an ethical person.
BttrThnOthrs	Being better than others.
Charitable	Being charitable, helping the needy.
Clean&Neat	Being clean and neat (personal care).
CommitCause	Being committed to a cause (e.g., environment, anti crime, anti drugs).
Confident	Being confident and assured.
ConfJudge	Being confident in my own judgment.
Conventional	Being conventional or traditional.
Creative	Being creative (e.g., artistically, scientifically, intellectually).
Curious	Being curious.
Disciplined	Being disciplined, following my intentions with behavior.
EmoCloseChild	Being emotionally close to my children.
EmoClosePart	Being emotionally intimate (close) with a romantic partner.
Fashionable	Being fashionable.
Good_w/Tech	Being good at working with mechanical objects and technology.
Attractive	Being good looking, attractive.
Happy	Being happy and content.
Competent	Being highly competent.
Honest	Being honest.
Humble	Being humble.
InControl	Being in full control of ones life.
InLove	Being in love.
Smart	Being intelligent or smart.
Rational	Being logical, rational.
Loyal	Being loyal.
PartSocGrp	Being part of a social group.
PhysAct	Being physically active.
PhysFit	Being physically fit.
PhysHlth	Being physically healthy, e.g., maintaining a healthy weight, eating nutritious foods.
Playful	Being playful, carefree, lighthearted, enjoying life.
Popular	Being popular, being in the center of things.
Practical	Being practical, having common sense.
PassionAbSmthing	Being really passionate about something.
Respected	Being respected by others.
Responsible	Being responsible, dependable.
Independent	Being self-sufficient, independent.
Spontaneous	Being spontaneous.
SuccInOccup	Being successful in my occupation.
TknCareOf	Being taken care of.
Unique	Being unique or different.
BuyThngs	Buying things I want.
ContPhysEnv	Controlling my physical environment.
ControlOthrs	Controlling others.
Recreation	Devoting time to amusements, recreation, entertainment, hobbies.
EntertainOthrs	Entertaining, amusing others.
Equality	Equality.
NatBeauty	Experiencing natural beauty.
Adventurous	Exploring, being adventurous.
FeelGoodSelf	Feeling good about myself.
FeelSafe	Feeling safe and secure.
FeelSatisfact	Feeling satisfied with one's life.
HigherMeaning	Finding higher meaning in life.
Education	Getting an education.
GrwingSpirit	Growing spiritually.
HavGdJob	Having a good job.
GoodMarry	Having a good marriage.
HaveMentor	Having a mentor, someone to guide me.
StabFamLife	Having a stable, secure family life (with my spouse or children, or both).
EasyLife	Having an easy and comfortable life.
ExcitngLife	Having an exciting, stimulating life.
Occupation	Having an occupation.
AthAbility	Having athletic ability.
ClsFriends	Having close friends.
$ $Descend	Having enough money to leave for my descendants.
FrmVals	Having firm values.
FlxbleVue	Having flexibility of viewpoint.
IntellectExper	Having intellectual experiences and conversations.
OthrsTrustU	Having other people trust you.
Othrs2RelyOn	Having others to rely on.
PpleToDoThingsWth	Having people to do things with.
Sex	Having sexual experiences.
Stability	Having stability in life, avoiding change.
Wisdom	Having wisdom, a mature understanding of life.
WorkILike	Having work I really like.
HelpOthrs	Helping others.
InflOthrs	Influencing, persuading others.
InspirOthrs	Inspiring others.
Justice	Justice and fairness.
ThngsInOrdr	Keeping things in order (my desk, office, house, etc.).
KeepToSelf	Keeping to myself, being private.
UpToDate	Keeping up to date with career-related knowledge.
KnowSelf	Knowing myself.
LearnArts	Learning and appreciating the arts.
Cls2Fam	Living close to my parents, siblings, grandparents.
ReligFaith	Maintaining religious faith.
Make$ $ $	Making a lot of money.
Decide4Othrs	Making decisions for others.
MakeFrnds	Making friends, drawing others near.
Mastery	Mastering what I set out to do.
ObeyParents	Obeying my parents.
AdvanDegree	Obtaining an advanced educational degree.
OvercomeFail	Overcoming failure.
PlsGod	Pleasing God.
PracReligTrad	Practicing religious traditions.
ProvideFamily	Providing for one’s family.
PursueIdeals	Pursuing my ideals.
RecHelpFmly	Receiving help from my parents, siblings, grandparents.
RespectEld	Respecting my elders.
OwnGuidelines	Setting and following my own guidelines.
SetGoodEx	Setting good examples for others.
ShareFeelings	Sharing my feelings with others.
TchOthrs	Teaching others.
AcceptSelf	To accept myself, other people, or things as they are.
AttendToDetails	To attend to details.
AvNotice	To avoid being noticed.
AvConflict	To avoid conflict with others.
AvEffort	To avoid effort or work.
AvHrtOthr	To avoid hurting (annoying, upsetting, etc.) others.
AvOthrs	To avoid other people.
AvRespons	To avoid responsibility.
Alert	To be alert or attentive.
Active	To be busy or active.
Efficient	To be efficient, not waste time.
OnTime	To be on time.
PhysAble	To be physically able to do my daily/routine activities.
SelfControl	To be self-controlled.
Selfless	To be selfless, to put others first.
TrueToSelf	To be true to myself, (not follow the crowd).
CarefulThink	To carefully think through decisions.
Communicate	To communicate or express myself.
DoQuickly	To do things quickly.
EnforceAccount	To enforce accountability.
InTuneEmot	To get in tune with my emotions.
GetRevenge	To get revenge (get even, get back, etc.).
BeCorrect	To get things right (accurate, correct).
Empathy	To have empathy for what others are feeling.
HvOthrsGiveMe	To have others give me what I want.
Manageable	To keep things manageable.
ListenOthrs	To listen to others.
Live4Today	To live for today.
PlsOthrs	To make others happy or to please others.
Plan	To make plans.
Procrast	To procrastinate.
Perfection	To strive for perfection.
AsLongAsNecess	To take as long as necessary and not hurry.
UndrstndPhysObj	Understanding how physical objects/systems work.

### Motive-sorting method

To measure judged similarity, we used a sorting task in which participants were asked to sort together items that went together, instead of using direct similarity ratings. Direct ratings of the similarity among all possible pairs of 161 motives would require an extremely large number of ratings ((161*(161–1))/2 = 12,880) and thus take an unrealistically long time. Even though the sorting task is difficult and time consuming (around 45 minutes), it takes far less time than 12,880 direct similarity ratings (assuming 3 seconds/rating, it would take 11 hours to make all the ratings).

The motive-sorting task was implemented as a Flash based program, developed by the first author, that ran inside a standard Web browser with the appropriate Flash plug-in. The sorting interface consisted of a list of the motives to be sorted, in a scrolling list, on the left hand side of the screen and a sorting area, which took up the remainder of the screen. Participants were told to sort the motives into groups on the basis of how similar the motives were to one another. Because the intent of this task is to figure out participants’ view of the important features, they were not told anything about what features were relevant, but simply told to put items together that they thought belonged together. This kind of instruction is typical in this context, so that one can uncover the distinctions that are important to participants, without biasing them. The exact instructions were: “Your aim is to sort the goals, seen in the left panel, into groups on the basis of how similar the goals are to one another. Keeping your own values aside, please sort these goals objectively on the basis of common themes, or topics, into which you see them falling. There are no right or wrong answers. You may form as many groups as you need, though we recommend keeping to no more than 30 groups. You can have as many goals in each group as you wish.”

We used the term goal because we thought that it would be easier for subjects to think about the content of the concept. Our intuition was that a concept that a participant has no interest in attaining for themselves (like religion for many people) is difficult to think of as a motive, since it has no desirable attraction for the participant. In contrast, characterizing the to-be-sorted statements as goals allows lay people to more easily recognize that others may have desires to seek a specified outcome and thus attend to its meaningful content in making sorting judgments.

Participants sorted the motives by dragging them from the list, one at a time, onto the sorting area. If a new motive was dragged from the list onto an empty spot in the sorting area, a new category box popped up to hold the motive. Additional motives could be dragged into this category box. Motives could be dragged from the list, or existing category boxes, to empty parts of the sorting area to form new category boxes. Thus, if participants decided that a motive did not belong in a category box, they could drag it to a different category or “drop” it on the sorting area to create a new category. When participants had sorted all the motives, the list would be empty.

The "I'm done" button became active once participants had sorted at least 90 of the motives, but participants could continue to sort until they had sorted all 161 motives. The program continuously recorded participants' sorting throughout the entire sorting task.

### Sample recruitment

Participants were recruited in several different ways. One large group of 235 participants was recruited from the USC Psychology Department Subject pool. A second group of 125 participants was recruited from regular visitors to the yourmorals.org website. This is a high traffic research website (9,000–15,000 visits per month) that recruits participants to fill out a wide range of personality and other types of psychological measures. A third group of 126 was recruited through Amazon’s Mechanical Turk. Finally, 3 other participants were recruited by the use of online ads through Google, Yahoo, and ASK.com. This research was approved by the University of Southern California University Park Institutional Review Board. Written informed consent was obtained from all subjects.

### Sample demographics

489 individuals sorted all 161 motives. Ages ranged from 18 to 70, there were 330 women and 159 men in the 489 individuals who sorted all 161 motives. All individuals in this sample resided in the USA.

## Results

In the following we cluster analyze the data from the 489 individuals who sorted all 161 motives. The overall sample of 489 is an unusually large sample for this kind of analysis. It should provide a fairly stable estimate of the perceived similarity among motives overall.

The sorting data was analyzed using hierarchical cluster analysis in the cluster analysis program ClustanGraphics [53]. We used the number of times each pair of items was sorted into the same category as a measure of similarity between the items. This sorting data was translated into a 161 X 161 matrix of co-occurrence scores, where the number in each cell represented the number of times the two items defining that cell were sorted together. Higher numbers mean that the pair of items was viewed as more similar. The matrix of proximities or similarities was then analyzed using a cluster analysis technique called Ward's [54] method or Increasing Sums of Squares. This method is also known as the “within-groups sum of squares or the error sum of squares (ESS)” method and is designed to optimize the minimum variance within clusters. It has been found to outperform other clustering methods in many cases [55].

### Overall solution

**Fig 1:**
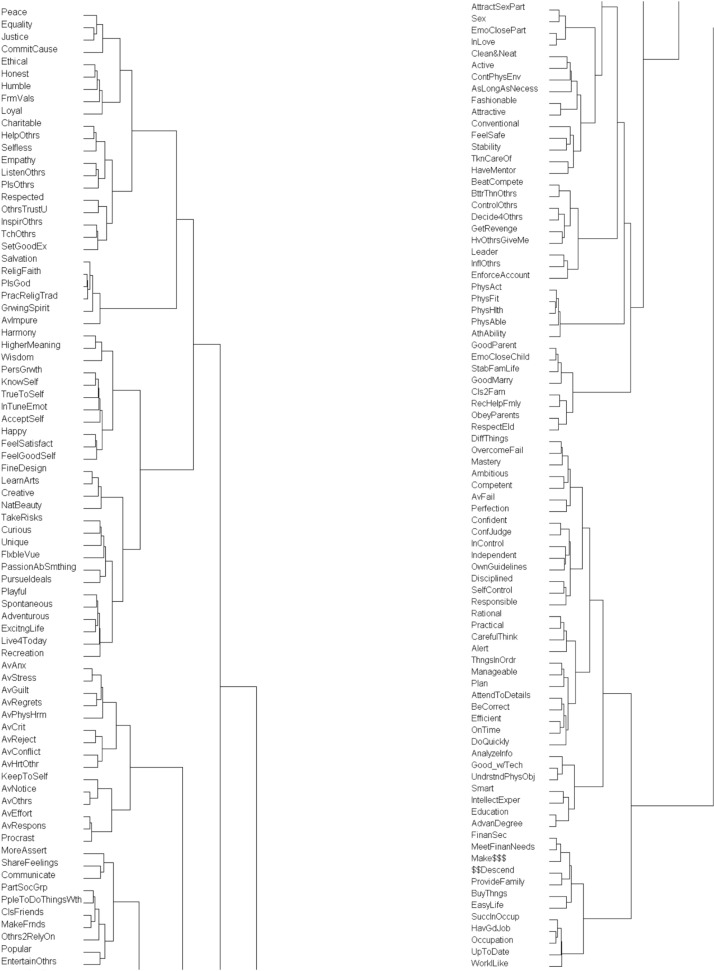
(A and B) Cluster Solution for Motive Taxonomy.

**Fig 2:**
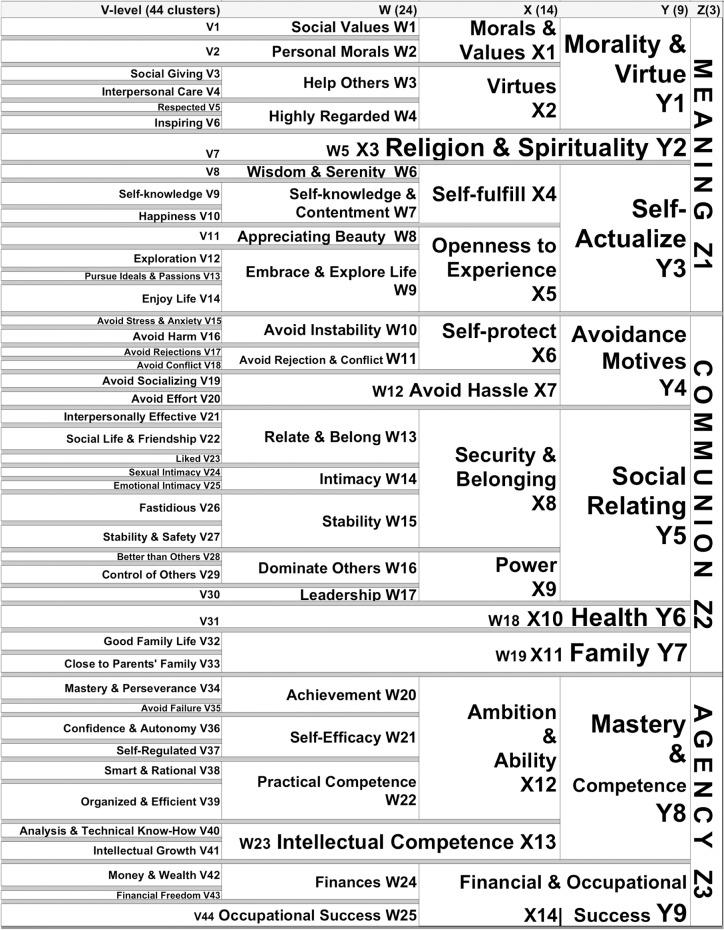
Labeled Clusters for Motive Taxonomy.

In general the results of the cluster analysis seem clearly understandable, both at the broad level and at the lower level of specific clusters. A detailed cluster diagram for this analysis can be found in Fig 1A and 1B. For those interested in a broader view of the structure of our results, or for a reference to what portion of our results may be of greatest interest, the major distinctions in our taxonomy are clearly illustrated in Fig 2. Our verbal description of the results will be an extensive examination for researchers working directly on related questions. We will separately examine each of three broad clusters as well as the lower level clusters that comprise them. In the discussion we will review a sample of the millennia of theories that our results support.

To develop the broader conceptual organization shown in Fig 2, the three senior authors of the paper started with the original cluster diagram (Fig 1A and 1B). The levels in the hierarchy that we identified were labeled, starting with Z at the highest level of abstraction, and then moving backwards in the alphabet. A number was assigned to each cluster within a level.

Inspection of the diagram indicated that it clearly divided into three distinct branches at the highest level. Next we turned to identifying the most concrete clusters. Our aim for this level was to retain the highest degree of detail possible such that each cluster was distinctly different in meaning from other clusters, yet within the cluster the items were coherent and similar to each other. This step involved a fair amount of discussion and debate among the three senior authors. In the end we identified and agreed upon 44 clusters at the most-concrete level (level V). The content of these 44 clusters is described in Table 2.

**Table 2:** 44 Motive Clusters and Their Contents.

Motive	Motive—Full description	V-level (44 clusters)
Peace	A world at peace.	Social Values V1
Equality	Equality.	
Justice	Justice and fairness.	
CommitCause	Being committed to a cause (e.g., environment, anti crime, anti drugs).	
Ethical	Being an ethical person.	Personal Morals V2
Honest	Being honest.	
Humble	Being humble.	
FrmVals	Having firm values.	
Loyal	Being loyal.	
Charitable	Being charitable, helping the needy.	Social Giving V3
HelpOthrs	Helping others.	
Selfless	To be selfless, to put others first.	
Empathy	To have empathy for what others are feeling.	Interpersonal Care V4
ListenOthrs	To listen to others.	
PlsOthrs	To make others happy or to please others.	
Respected	Being respected by others.	Respected V5
OthrsTrustU	Having other people trust you.	
InspirOthrs	Inspiring others.	Inspiring V6
TchOthrs	Teaching others.	
SetGoodEx	Setting good examples for others.	
Salvation	Achieving salvation.	Religion & Spirituality V7
ReligFaith	Maintaining religious faith.	
PlsGod	Pleasing God.	
PracReligTrad	Practicing religious traditions.	
GrwingSpirit	Growing spiritually.	
AvImpure	Avoiding impure acts.	
Harmony	Achieving harmony and oneness (with self and the universe).	Wisdom & Serenity V8
HigherMeaning	Finding higher meaning in life.	
Wisdom	Having wisdom, a mature understanding of life.	
PersGrwth	Achieving personal growth.	Self-knowledge V9
KnowSelf	Knowing myself.	
TrueToSelf	To be true to myself, (not follow the crowd).	
InTuneEmot	To get in tune with my emotions.	
AcceptSelf	To accept myself, other people, or things as they are.	
Happy	Being happy and content.	Happiness V10
FeelSatisfact	Feeling satisfied with one's life.	
FeelGoodSelf	Feeling good about myself.	
FineDesign	Appreciating fine design.	Appreciating Beauty V11
LearnArts	Learning and appreciating the arts.	
Creative	Being creative (e.g., artistically, scientifically, intellectually).	
NatBeauty	Experiencing natural beauty.	
TakeRisks	Being able to take risks.	Exploration V12
Curious	Being curious.	
Unique	Being unique or different.	
FlxbleVue	Having flexibility of viewpoint.	
PassionAbSmthing	Being really passionate about something.	Pursue Ideals & Passions V13
PursueIdeals	Pursuing my ideals.	
Playful	Being playful, carefree, lighthearted, enjoying life.	Enjoy Life V14
Spontaneous	Being spontaneous.	
Adventurous	Exploring, being adventurous.	
ExcitngLife	Having an exciting, stimulating life.	
Live4Today	To live for today.	
Recreation	Devoting time to amusements, recreation, entertainment, hobbies.	
AvAnx	Avoiding anxiety.	Avoid Stress & Anxiety V15
AvStress	Avoiding stress.	
AvGuilt	Avoiding feelings of guilt.	Avoid Harm V16
AvRegrets	Avoiding regrets.	
AvPhysHrm	Avoiding physical harm.	
AvCrit	Avoiding criticisms from others.	Avoid Rejections V17
AvReject	Avoiding rejection by others.	
AvConflict	To avoid conflict with others.	Avoid Conflict V18
AvHrtOthr	To avoid hurting (annoying, upsetting, etc.) others.	
KeepToSelf	Keeping to myself, being private.	Avoid Socializing V19
AvNotice	To avoid being noticed.	
AvOthrs	To avoid other people.	
AvEffort	To avoid effort or work.	Avoid Effort V20
AvRespons	To avoid responsibility.	
Procrast	To procrastinate.	
MoreAssert	Be less shy or more assertive.	Interpersonally Effective V21
ShareFeelings	Sharing my feelings with others.	
Communicate	To communicate or express myself.	
PartSocGrp	Being part of a social group.	Social Life & Friendship V22
PpleToDoThingsWth	Having people to do things with.	
ClsFriends	Having close friends.	
MakeFrnds	Making friends, drawing others near.	
Othrs2RelyOn	Having others to rely on.	
EntertainOthrs	Entertaining, amusing others.	Liked V23
Popular	Being popular, being in the center of things.	
AttractSexPart	Being able to attract a sexual partner.	Sexual Intimacy V24
Sex	Having sexual experiences.	
EmoClosePart	Being emotionally intimate (close) with a romantic partner.	Emotional Intimacy V25
InLove	Being in love.	
Clean&Neat	Being clean and neat (personal care).	Fastidious V26
Active	To be busy or active.	
ContPhysEnv	Controlling my physical environment.	
AsLongAsNecess	To take as long as necessary and not hurry.	
Fashionable	Being fashionable.	
Attractive	Being good looking, attractive.	
Conventional	Being conventional or traditional.	Stability & Safety V27
FeelSafe	Feeling safe and secure.	
Stability	Having stability in life, avoiding change.	
TknCareOf	Being taken care of.	
HaveMentor	Having a mentor, someone to guide me.	
BeatCompete	Beat people in a competition.	Better than Others V28
BttrThnOthrs	Being better than others.	
ControlOthrs	Controlling others.	Control of Others V29
Decide4Othrs	Making decisions for others.	
GetRevenge	To get revenge (get even, get back, etc.).	
HvOthrsGiveMe	To have others give me what I want.	
Leader	Being a leader, being in charge.	Leadership V30
InflOthrs	Influencing, persuading others.	
EnforceAccount	To enforce accountability.	
PhysAct	Being physically active.	Health V31
PhysFit	Being physically fit.	
PhysHlth	Being physically healthy, e.g., maintaining a healthy weight, eating nutritious foods.	
PhysAble	To be physically able to do my daily/routine activities.	
AthAbility	Having athletic ability.	
GoodParent	Being a good parent (teaching, transmitting values).	Good Family Life V32
EmoCloseChild	Being emotionally close to my children.	
StabFamLife	Having a stable, secure family life (with my spouse or children, or both).	
GoodMarry	Having a good marriage.	
Cls2Fam	Living close to my parents, siblings, grandparents.	Close to Parents' Family V33
RecHelpFmly	Receiving help from my parents, siblings, grandparents.	
ObeyParents	Obeying my parents.	
RespectEld	Respecting my elders.	
DiffThings	Accomplishing difficult things, overcoming challenges.	Mastery & Perseverance V34
OvercomeFail	Overcoming failure.	
Mastery	Mastering what I set out to do.	
Ambitious	Being ambitious, hard-working.	
Competent	Being highly competent.	
AvFail	Avoiding failure.	Avoid Failure V35
Perfection	To strive for perfection.	
Confident	Being confident and assured.	Confidence & Autonomy V36
ConfJudge	Being confident in my own judgment.	
InControl	Being in full control of ones life.	
Independent	Being self-sufficient, independent.	
OwnGuidelines	Setting and following my own guidelines.	
Disciplined	Being disciplined, following my intentions with behavior.	Self-Regulated V37
SelfControl	To be self controlled.	
Responsible	Being responsible, dependable.	
Rational	Being logical, rational.	Smart & Rational V38
Practical	Being practical, having common sense.	
CarefulThink	To carefully think through decisions.	
Alert	To be alert or attentive.	
ThngsInOrdr	Keeping things in order (my desk, office, house, etc.).	Organized & Efficient V39
Manageable	To keep things manageable.	
Plan	To make plans.	
AttendToDetails	To attend to details.	
BeCorrect	To get things right (accurate, correct).	
Efficient	To be efficient, not waste time.	
OnTime	To be on time.	
DoQuickly	To do things quickly.	
AnalyzeInfo	Being able to analyze and synthesize information.	Analysis & Technical Know-How V40
Good_w/Tech	Being good at working with mechanical objects and technology.	
UndrstndPhysObj	Understanding how physical objects/systems work.	
Smart	Being intelligent or smart.	Intellectual Growth V41
IntellectExper	Having intellectual experiences and conversations.	
Education	Getting an education.	
AdvanDegree	Obtaining an advanced educational degree.	
FinanSec	Achieving lifetime financial security.	Money & Wealth V42
MeetFinanNeeds	Being able to meet my financial needs.	
Make$ $ $	Making a lot of money.	
$ $Descend	Having enough money to leave for my descendants.	
ProvideFamily	Providing for ones family.	
BuyThngs	Buying things I want.	Financial Freedom V43
EasyLife	Having an easy and comfortable life.	
SuccInOccup	Being successful in my occupation.	Occupational Success V44
HavGdJob	Having a good job.	
Occupation	Having an occupation.	
UpToDate	Keeping up to date with career-related knowledge.	
WorkILike	Having work I really like.	

The next step was to identify the intervening structure between the most concrete and most abstract level of motives. In a cluster diagram, similarity is represented by the horizontal distance (or length) of a branch between leaves. To identify similar levels of construal we drew vertical lines to mark the horizontal branches at even intervals (Fig 1A and 1B). This gave us a visual guide to common similarity distances. We tried different numbers of dividing lines to see which gave us the most meaningful and coherent set of divisions.

In the end, three intermediate-level lines each touched (or neared) a particularly high number of clusters throughout the hierarchy. Face validity confirmed that clusters at the same vertical marker (aka, clusters at a similar horizontal distance from the origin) were similar in construal level. Moreover, at each of the intermediate levels, the resulting clusters were meaningful and distinct from others at the same level.

Following the horizontal connectors in Fig 1A and 1B, the diagram seamlessly connects a cluster at one construal level both to the more concrete clusters that constitute it and to a more abstract cluster of which it is a part. Power, for instance, may more concretely entail dominating others and is, more abstractly, a type of Social Relating.

### Hierarchical structure by construal level: The structure and theory

Five hierarchical levels of construals emerged from the taxonomy as distinct and coherent (Fig 2). The three highest cluster levels (X, Y, & Z) are quite abstract and particularly relevant to existing theory about the type and nature of high-level motives. The two lowest levels (V & W) are the most concrete.

At the broad levels there seems to be a strong correspondence between our results and other theoretical accounts of the structure of human motives. At the top, our hierarchy branches into three clusters: Meaning (Z1), Communion (Z2), and Agency (Z3). This is our “Z-level”. This discovery of meaning motivations as being distinct from agency and communion is unique to this work.

#### Meaning (Z1) motives in theory

Pursuit of meaning or the purpose of life has spun theories reaching back into the ages from philosophy, religion, and a multitude of scientific disciplines. We identify the motives to which these theories of meaning and living give rise.

To Plato, the meaning of life was the attainment of the highest form of knowledge. And to know thy self was but the start [56]. The common Greek expression “virtue is knowledge" highlights the strong relationship for them between wisdom and virtue.

The word “virtue” in Greek, “*arête*”, means to reach one’s highest unique potential. A similar modern concept is self-actualization, at the peak of Maslow’s hierarchy of needs. Included is the need for morality, creativity, spontaneity, and experiencing (esp. Peak Experiences).

Aristotle wrote that everything done is for a goal and most goals are really only means to higher-order goals [57]. Goal pursuit itself is a “good.” And the goal of all goals, the highest aim, is happiness (called *eudaimonia*), which is more precisely translated as “human flourishing” by “doing things well.”

These themes are also found in the work of Martin Seligman on well-being: 1) Engagement, which can be experienced in even mundane tasks by utilizing one’s highest strengths or virtues [58]. 2) Meaning, to belong to and serve something greater than one’s self. 3) Achievement, to advance from the starting point of a motive, also know as goal pursuit. The remaining elements are found later in our taxonomic discussion: 4) positive emotion and 5) relationships [59].

The rise of the Judeo-Christian belief system ushered in the more prescriptive definition of virtue (e.g. “thou shalt not lie”) and morals that is the lay concept of today. Life’s purpose in Western and Middle Eastern religions is to live a good life prescribed by religious guidelines, a connection with God, and/or to earn an appealing afterlife.

In the east, the Hindu religion provided its own taxonomy of meaning motives known as the *purusharthas*: The first category, *Kama*, involves wishing, desire, love, and sensual pleasure. Wealth, prosperity and glory comprise the second category, *Artha*. *Dharma* includes righteousness, duty, morality, virtue, and ethics. And the fourth Hindu aim, *Moksha*, is liberation (from Saṃsara, the cycle of reincarnation).

Buddhism does not address “the meaning of life” but the potential of human life to end suffering. There is a distinction in Buddhism between adaptive desires (Chanda) and the maladaptive (Taṇhā) desires that lead to suffering. Chanda is the desire for well-being, self-improvement, and goodness. The latter leads to effort and action, and is founded upon intelligent reflection and wisdom–note the congruence here with the Greek philosophy.

#### Meaning (Z1) in focus

Morality & Virtue (Y1), Religion & Spirituality (Y2), and Self-Actualization (Y3) all cluster together and represent a general need for meaning or purpose in life. Morality & Virtue include Social Values (W1, e.g., peace and justice, loyalty), Personal Morals (W2, e.g. be ethical and honest), as well as virtues such as Help Others (W3, e.g. charitable, listening) and being Highly Regarded (W4, e.g. being trusted and Inspiring (V6)). We recognize that Help Others (W3) could also fit, conceptually, in the Communion cluster. However, our empirical results place it with Meaning (Z1), where it also fits with other virtues. Religion & Spirituality is most conceptually distinct in that it maintains its independence through the remaining four levels (Y2/X3/W5/V7) and includes motives such as maintaining faith, growing spiritually, and achieving salvation. Also found in this category is avoiding impure acts.

Harkening back to the Greek definition of virtue, the Self-Actualization [8,60] cluster (Y3) contains needs for Self-Fulfillment (X4) and Openness to Experience (X5). The Self-Fulfillment cluster is a veritable cocktail of healthy hedonism. Among the related motives are such things as Wisdom and Serenity (W6/V8), Self-knowledge (V9), and Happiness (V10). Openness to Experience (X5) includes lower-level motive clusters like Appreciating Beauty (W8/V11), Exploration (V12), Pursuing Ideals & Passions (V13), and Enjoying Life (V14). Some of these components may also be similar to Self-determination theory’s Autonomy (but we think this construct has a better fit below, as we will soon discuss).

#### Agency (Z3) & communion (Z2) motives in theory

An extensive literature draws a distinction between Agentic and Communal orientations (e.g., [61–63]) (often termed Agency versus Communion). An individual with an Agentic orientation tends to be focused on individual achievement and activities, which is represented in our Agency (Z3) cluster. In contrast, a Communal orientation is more focused on the group or community and involves interactions with and caring for others. This maps largely onto our Communion (Z2) cluster. A major difference between cluster Z2 and the typical view of communal orientation is that Z2 includes avoidance motives and security concerns. However, most of the avoidance motives are interpersonal in nature.

The Agentic-Communal distinction is also similar, at least roughly, to those presented in Self-determination Theory. Self-determination theory [64] argues that there are three basic psychological needs that people try to satisfy: Competence, Relatedness, and Autonomy. Our hierarchy illuminates how these theories are related by suggesting that Relatedness is akin to Communion while Competence and Autonomy are aspects of Agency. Specifically, in our hierarchy: Communion (Z2) includes, e.g. Social Relating (Y5) and Family (Y7). Under Agency (Z3) is found Mastery & Competence (Y8), which includes Confidence & Autonomy (V36), and Self-Regulation (V37, most similar to Deci & Ryan’s Competence need), and intellectual and practical competency clusters (W22 & W23).

#### Communion (Z2) in focus

Communion (Z2) includes four highly abstract clusters: Avoidance Motives (Y4), Social Relating (Y5), Health (Y6), and Family (Y7). The first two clusters in Communion can be thought of as approaching (Social Relating, Y5) and avoiding (Y4: Avoidance Motives) people. Social Relating (Y5) has to do with various aspects of social interaction and relating to others including Security & Belonging (X8) as well as Power (X9). Taking a more concrete look, Security and Belonging (X8) is comprised of motives to Relate & Belong (W13), Intimacy (W14), and Stability (W15). Motives to Relate & Belong include motives for communicating and sharing feelings (Interpersonal Effectiveness, V21), having a Social Life & Friendship (V22), and being Liked (V23). Intimacy (W14) consists of two closely related clusters, one having to do with attracting a sex partner and having sexual experiences (Sexual Intimacy, V24) and the other with being in love and emotionally close to a partner (Emotional Intimacy, V25). Stability (W15) is comprised of both Stability & Safety (V27) motives as well as a cluster that contains a set of items related to being clean and neat, as well as being attractive (Fastidiousness, V26).

The next abstract or theory-level cluster under Social Relating (Y5) is Power (X9). Power motives include motives for winning over or being Better than Others (V28), a second cluster concerning Control of Others (V29) and a final cluster concerned with leading and influencing others (Leadership, W17). Although Power (X9) has a clear hierarchical structure, its location in the Communion cluster, instead of the Agency cluster does seem to be an anomaly. One possible reason is that the motives in the Communion cluster, including Power, are highly *inter*-personal, whereas the motives in the Agency cluster are largely *intra-*personal.

Avoidance Motives (Y4) include Avoid Harm (V16), both physical and emotional, Avoid Stress & Anxiety (V15), Avoid Rejection (V17), Avoid Conflict (V18) including hurting others, and Avoid Socializing (V19). Note these are all social in nature although Avoid Socializing next clusters with Avoid Effort (V20) to become a part of Avoid Hassles (X7/W12).

There is then a coherent cluster of items concerned with being physically able, health, and athleticism. Health is a branch that maintains its independence through four levels of the hierarchy (Y6/X10/W18/V31). Here it clusters under Communal Motives (Z2).

The final set of clusters in the Communion (Z2) branch deal with various aspects of Family (Y7). Good Family Life (V32) includes the motives being a good parent, being close to children, and having a stable family life and marriage. Then there is a cluster that deals more with one’s relationship to one’s own parents and siblings (Close to Parent’s Family, V33): being close to family and receiving help from family, and obeying parents and respecting elders. These two lowest-level clusters then join and the resulting cluster (Family, W19/X11/Y7) thereafter maintains its independence until it meets the Z-level under the Communal branch.

#### Agency (Z3) in focus

The motives located in the bottom branch of our hierarchy clearly relate to Agency. It includes Mastery & Competence (Y8) and Financial & Occupational Success (Y9/X14).

The former includes Ambition and Ability (X12) and Intellectual Competence (X13/W32). Ambition & Ability consists of several different clusters. Starting from the most concrete instrumental level we first see a motive cluster concerned with Mastery & Perseverance (V34) followed by motives to Avoid Failure (V35). These both have to do with Achievement (W20). Next, Self-Efficacy (W21) includes clusters about being confident, in control of the environment, independent (Confidence & Autonomy, V36), disciplined and self-controlled (Self-Regulated, V37).

Next, there is a cluster concerned with thinking and being rational and practical (Smart & Rational, V38). Then there is a cluster dealing with conscientiousness, a desire for things in order and a desire to be correct, efficient and on time (Organized & Efficient, V39). These combine to form Practical Competence (W22).

Following Ambition and Ability (X12) we see a cluster concerned with Intellectual Competence (X13/W23) related to understanding physical objects and systems (Analysis and Technical Know-How, V40), and being smart and having intellectual experiences, and being highly educated (Intellectual Growth, V41). Ambition and Ability (X12) and Intellectual Competence (X13) combine to form a broader category we term Mastery & Competence (Y8).

The final large cluster is concerned with Financial and & Occupational Success (Y9/X14), which includes subclusters involving Finances (W24), composed of two further subclusters involving Money/Wealth (V42) and Financial Freedom (V43), and a subcluster concerned with having a good job and being successful in it (Occupational Success, W25/V44).

### Summary

In general, with some minor deviations, this presents a remarkably coherent view of the structure of human motivation. The broadest level of the taxonomy has strong parallels with broad distinctions made by other theorists, and the most specific level of the hierarchy exhibits coherent clusters of motives that systematically join together into the higher-level structures. Work on the Big Five suggests that while the higher, more abstract levels are theoretically interesting, predictive power increases as one moves to lower, concrete, levels (see Use of the Taxonomy in Predicting Behavior in the discussion section).

## Discussion

We identified five major, conceptually meaningful cluster-levels in our taxonomy. At the highest level we identified three major clusters: Meaning (Z1), Communion (Z2), and Agency (Z3). As we discussed in the results and in the discussion below, these three clusters map onto major distinctions that have been made previously throughout the literature. However, our meaning cluster seems to be a unique grouping in the literature on the structure of human motives.

### Comparison with previous categorizations of human motivators

Comparison with previous semantically based categorizations of the motivational domains reveals some overlap at various construal levels.

#### Agency vs. communion

This distinction, also called Competence vs. Relatedness, has a notable history in the literature. Braithwaite and Law’s interviewees made the same distinction as our participants here when they created their Goal and Mode Values Inventories [46]. The same distinction was found in Ford and Nichols’s categorization [29]. Moreover, this distinction parallels work on cultural differences in values, specifically the difference between Individualist and Collectivist values (e.g., [65,66]). It is central in the work on personal orientation identified as Agency versus Communion [61,62]. Thus, both laypeople and theoreticians seem to perceive being competent and agentic in the world as very different from interacting with others and relatedness.

#### Murray’s needs

There are also some clear parallels between the results in our study and Murray’s classic conceptualization of needs [10]. For instance, Murray’s Affiliation looks similar to our Social Life & Friendship (V22) cluster in the present study. Murray’s Dominance is similar to this study’s Leadership (W17) cluster, which includes, “control of environment,” “persuading others,” “decisions for others,” “control over others,” “leader,” and “setting examples.”

Physical Ability is similar to this taxonomy’s Health (Y6) cluster. Economic Ability is similar to Finances (W24). Erotic Ability—the ability to please, attract and excite the opposite sex; to love and be loved—is parallel to Intimacy (W14). This taxonomy’s Intellectual Competence (X13/W23) cluster seems to be tapped into by three of Murray’s Abilities or Achievements: Intellectual Ability; Scientific Ability; and Theory-Creative Ability—the ability to construct explanatory concepts in science, to devise good hypotheses. Murray’s Aesthetic Ability is similar to our Appreciating Beauty (W8/V11).

#### Maslow’s hierarchy of needs

There are a number of parallels between Maslow’s theoretical classifications of human motivators [8,60] and our taxonomy. For example, Maslow’s Physiological (or Biological) needs are similar to this study’s Health (Y6) and Sexual Intimacy (V24). His Security and Safety needs are captured by our Stability (W15) and Avoidance Motives (Y4), which, in all sub solutions do cluster together. Maslow’s Affiliation (or Attachment) needs tap into Interpersonal motives such as having a Social Life & Friendship (V22) and to be Liked (V23). His cognitive needs resemble our taxonomy’s Intellectual Competence (X13/W23), and creativity-related motives, the latter of which cluster in our taxonomy under the highest of Maslow’s needs: Self-Actualization (Y3). This includes motives for Self-Fulfillment (X4) including Self-knowledge, and Openness to Experience (X5).

Although Maslow’s taxonomy is hierarchical, that hierarchy is not based on conceptual relationships. Rather it is based on a hierarchy of need satisfaction. Maslow argued that motives lower in the hierarchy would be satisfied first, and only when they had been satisfied, would the individual pursue higher order motives. Thus, pursuit of Self-actualization, the pinnacle of his hierarchy, would only occur once lower order motives had been satisfied. In contrast, our hierarchy is based on conceptual relationships, with higher order motives being more abstract and encompassing a wider range of more specific motives.

#### Braithwaite and law

Braithwaite and Law [46] provide broad motive categories. There is a distinction between Agency vs. Communion as discussed above. Under communion, their category “Secure and Satisfying Interpersonal Relationships,” included “Mature love,” “True friendship,” “Personal support,” “Security for loved ones,” and “Acceptance by others.” However, this combines distinct motivational domains that, in our taxonomy, comprise at least 5 separate clusters.

#### Schwartz’s values circumplex

The most ambitious study to date of the structure of human motivation is Schwartz’s [36,37] Value Circumplex. Schwartz has studied the structure of human values across numerous countries. Many of Schwartz’s values have parallels with the clusters we find in our analyses. Using multi-dimensional scaling (MDS) they consistently find 10 major groups within the dimensions identified by the MDS (recently divided into 19 [38]: Self-Direction (Thought, Action), Stimulation, Hedonism, Achievement, Power (Dominance, Resources), Security (Personal, Societal), Tradition, Conformity (Rules, Interpersonal), Humility, Universalism (Concern, Tolerance), Benevolence (Caring, Dependability).

A visual inspection of the MDS plots shows that these categories seem to fall along two broad dimensions: Self-Enhancement vs. Self-Transcendence and Openness to Change vs. Conservation. The plots also show that regions on one side of the wheel group into Personal Focus (similar to Agency) and the other side into Social Focus (similar to Communion). Alternatively, one side of the wheel looks to group along Anxiety-Free values and Self-Protection Anxiety/Avoidance related values. The main interest for Schwartz is not the hierarchical structure but the dynamic structure of congruence and conflict among motives.

Our taxonomy of motives and Schwartz’s values circumplex have different origins and intentions. We see the differences between the structures as complementary and will summarize some of them here. First, Schwartz’s values are largely *theoretically derived* and consistent with empirical results (e.g., [36,38] whereas our taxonomic structure is *empirically derived* and consistent with theory. Second, our work uses a meaning-based structure of similarity judgments while Schwartz’s work relies on importance judgments. Importance judgments, in line with Lewin’s distinctions, capture the strength of wanting, but not motive content. Schwartz’s circumplex captures meaning only indirectly by making additional theoretical assumptions about congruence and incongruence among values that follow from adaptive engagement or disengagement with motives made feasible or unattainable by situational affordances. The latter is used so that values can be arranged such that congruent values are adjacent and conflicting values opposite one another on a circumplex. Thus, ours is a taxonomy in which motive contents are measured directly, while the contents and structure of Schwartz’s values are inferred responses to the constraints and affordances of the human condition.

Finally, Schwartz focuses on values whereas we focus on motives. There are two key differences between values and motives. Values have a sense of being tied to morality and ethics. There is a sense that they are normatively desirable, whereas the idea of motives is morally neutral. Consistent with Schwartz’s work, the taxonomy also represents motives relevant to morality and meaning in life, and these cluster together in the abstract (Meaning Z1) or with their means of attainment in the concrete, as discussed above. However, these motives are members, not the focus of our taxonomy. The second key difference between values and motives is that values are trait like “guiding principles” for one’s life. In this way, values are more descriptive of what “should” motivate one while motives are what “does”. Motives are more reflective of the situation, in adaptive response to it, whereas values are supposed to be more reflective of the person and their own internal compass.

A comparison of our meaning-based structure with Schwartz’s importance-based structure is illuminating. For instance, in the Schwartz circumplex, Power and Universalism (~ Y1 Morality & Virtue) are located on opposite sides of the wheel indicating a strong oppositional relationship. In contrast, our total solution indicates that there is *no* relationship between power and morality motives. Power, and its subcluster Leadership (V30), is in an entirely separate branch from Morality & Virtue: Communion (Z2) and Meaning (Z1), respectively. Comparison of a structure that captures meaning and a structure that captures values would allow us to see how the structure of our values differs from the conceptual structure of our motives. This might lend insight into what our culture *is* as compared to what it *could* be.

#### Self-determination theory

At the broadest level there appears to be a strong correspondence between our results and the distinctions proposed in Self-determination theory (SDT) [64]. SDT argues that there are three basic psychological needs that people try to satisfy: Competence, Relatedness, and Autonomy. The extent to which these three needs are achieved or thwarted has important implications for physical and psychological well-being. Deci and Ryan and their colleagues have developed an extensive body of theoretical and empirical work investigating the nature of these needs, how they are influenced by the environment and other people, and the implications of the satisfaction or thwarting of these needs for human well-being.

Competence refers to a motivation or psychological need to be able to effectively influence the environment and to attain rewards and avoid punishments within it. Relatedness refers to a desire to be loved and cared for by others, to feel connected. Autonomy concerns a sense of volition, of being able to behave freely in line with one’s integrated sense of self. It is not simply having an internal locus of control. Relatedness with others and Competence in the world are clearly represented in the current structure (see Fig 1A and 1B). This distinction between Competence and Relatedness is similar to the frequently identified difference between Agentic (Z3) and Communal (Z2) orientations (e.g., [61,62]), which we find to be major branches in our taxonomy. Autonomy is also strongly represented, particularly within some aspects of our Meaning (Z1) cluster.

#### Ozer

Reisz et al [43], in a paper examining the relationship between personality traits and personal goals, briefly describe a 3 level hierarchical goal taxonomy, in which 96 goal categories are organized into three levels, with the highest level consisting of eight broad goal categories: Academic/Occupational, Social Relationships, Financial Concerns, Health and Fitness, Organization, Affect Control, Independence, and Moral or Religious. Unfortunately, the details of how the taxonomy was constructed are not presented.

There are numerous similarities with the current taxonomy, and many of the important distinctions we make can also be found in their taxonomy, both at the broadest level and in terms of the distinctions or subdomains we both identify. However, we have more levels in our taxonomy (five), including a high level distinction between Agency and Communion. In contrast, the top level of their taxonomy is at the level of their 8 broad categories, which are not organized into any higher order structure, such as Agency, Community, and Meaning. We also note that we have a much larger number of motives, 161, than they do.

#### Reiss

Reiss and Havercamp [67,68] developed a 128 item measure (Reiss Profile of Fundamental Goals and Motivational Sensitivities) to assess 16 motivational domains, each of which was hypothesized to be a basic or fundamental motive. Each domain was measured by 8 items. However, this was not an attempt to create a hierarchical taxonomy of human motives, but only provided one level of organization. Most of the motivational domains he identified correspond to motives in our taxonomy. His 16 motivational domains are: *“Power*, Desire to influence (including leadership; related to mastery); *Curiosity*, Desire for knowledge; *Independence*, Desire to be autonomous; *Status*, Desire for social standing (including desire for attention); *Social contact*, Desire for peer companionship (desire to play); *Vengeance*, Desire to get even (including desire to compete, to win); *Honor*, Desire to obey a traditional moral code; *Idealism*, Desire to improve society (including altruism, justice); *Physical exercise*, Desire to exercise muscles; *Romance*, Desire for sex (including courting); *Family*, Desire to raise own children; *Order*, Desire to organize (including desire for ritual); *Eating*, Desire to eat; *Acceptance*, Desire for approval; *Tranquility*, Desire to avoid anxiety, fear; *Saving*, Desire to collect, value of frugality.” (Reiss, [67], p. 187)

#### Evolutionary/Functional approaches

Recently several researchers [40,42] have taken an evolutionary and functional approach to conceptualizing motives. Based on her analysis Bugental [40] has identified five major domains or tasks in human life and the broad motivational systems that have evolved to address each of these tasks. The domains and their related tasks are: Attachment (safety maintenance), Coalitional group formation and maintenance (defending, acquiring shared resources and territory), Mating (selecting and maintaining/protecting access to a high value mate), Reciprocity (maximizing joint outcomes for functional equals), and Hierarchical power (optimizing welfare and balance of control between those of unequal power). Each of these domains is clearly represented in the current taxonomy. Note again that Bugental does not outline any hierarchical or conceptual relationship among these motives.

Kenrick, Griskevicius, Neuberg, and Schaller [42] took an evolutionary and functional approach to reconceptualizing Maslow’s [8] hierarchy of needs. Maslow’s original hierarchy, as noted above, was not a conceptual hierarchy, but instead represented the ordering in which motives needed to be satisfied and the dependency among motives. For example, Maslow suggested that basic bodily needs had to be satisfied before individuals would pursue higher order needs and most notably that they had to satisfy motives lower in the hierarchy before the ultimate motive of self-actualization could be pursued. Kenrick et al, while recognizing that some motives take precedence over others when activated (e.g., safety needs or food needs), argued for a more overlapping system and more flexible priorities. They also suggested that motives followed a developmental trajectory, so that motives such as mating and parenting would only come on line at the appropriate developmental period. Finally, they reconceptualized the motives in Maslow’s hierarchy, suggesting that the major motivational categories would be: Immediate Physiological Needs, Self-Protection, Affiliation, Status/Esteem, Mate Acquisition, Mate Retention, and Parenting. All of these high level motives are clearly represented in our taxonomy.

Bernard and Lac [41] developed a questionnaire measure of individual differences in the strength of 15 motivational dimensions, based on their evolutionary based theory of human motivation. Similar to Bugental, they argued that domain specific motives evolved to meet the major challenges that humans faced in the environments of evolutionary adaptedness (EEAs). They further suggested that there would be individual differences in the potential expression of each motivation as a function of genetic and environmental influences. The 15 motives they measured are: Environmental Inquisitiveness (explore environment to evaluate hazards and resources), Illness Avoidance, Threat Avoidance, Aggression (to acquire and control resources), Interpersonal Inquisitiveness (explore the social environment), Appearance (compete for status on basis of physical appearance), Mental (compete for status on basis of mental attributes), Physical (compete for status on basis of physical capabilities), Wealth (compete for status on basis of material resources), Commitment (to mate and close kin, intimate attachments), Altruism (transfer resources to kin without expectation of return), Social Exchange (reciprocal exchange of resources), Legacy (transfer of resources to institutions that benefit non-kin), and Meaning. Although Bernard and Lac (p. 50) have argued that theoretically the motives are independently evolved strategies, their empirical results suggest that some of their dimensions are interrelated in meaningful ways: for example, competitive/status motives are inter correlated as are the cooperative motives. Thus, there may be some hierarchical structure, although they leave that to future work.

All of these evolutionary/functional approaches have several important differences from the current approach. First, all of these researchers focus on broad motive domains. They do not attempt to determine whether there is a more hierarchical structure of broader and more specific domains, and if so, what that structure might be. Second, all three analyses are based almost entirely on a functional and conceptual analysis of the major domains of human life. Neither group was concerned with examining the hierarchical conceptual structure of human motives.

### Comparison with idiographic techniques

The taxonomy we present here offers a number of advantages over idiographic approaches. It is easier for both researcher and participant. For the participant, merely sorting or rating existing items is faster and easier than self-generating them. Moreover, once these items are generated, they must be coded by researchers who wish to find out what is important to people in their particular context. Thus, using this taxonomy means no coding is necessary in order to compare motive or goal contents.

In addition, the quality of motives is a problem for the idiographic technique. Unfortunately, self-generated motives tend to be repetitive. People will rephrase the same motive to fill space, or list sub-goals that are really just lower-level means by which to accomplish the same motive. Further, self-generated motives are more sensitive to current contexts–the apparent topic of the study or what Ss are currently grappling with today. The taxonomy clusters, on the other hand, cue a life-balanced breadth of associations so that you find out what’s important to people–not just what they can think of right then. Furthermore, what’s important is only half the story. Often, what’s unimportant can also be valuable information–information that will be harder to gather as it is harder to remember or even imagine.

This taxonomy provides the foundation for a domain-general measure of the role of various motives in important life decisions. Not only does it provide for ease of measurement across a wide array of life domains, but because we are using a common set of motives we can easily compare the importance of different motives across a variety of life choices.

In addition to facilitating our ability to make comparisons across life domains, it also facilitates our ability to make comparisons across individuals. Many of the researchers who have studied the role of various goal or motive constructs in human behavior have taken a somewhat idiographic approach (e.g, [4,5,7]). They typically ask people to write down their important life goals, which are then coded. This is both time and labor intensive and makes comparison across individuals difficult. In contrast, our comprehensive taxonomy makes it relatively straightforward to compare individuals using a common set of items.

### Use of the taxonomy in predicting behavior

Since developing the taxonomy presented here, we have used it in many studies to predict behavior in several different domains. Our general procedure is to first choose a level of the hierarchy at which we want to measure motives, such as at the 44-cluster level or the 14 cluster level. We then ask participants to indicate the importance of the motives by using a Q-sort type procedure in which they sort the motives into a forced-choice, quasi-normal distribution of categories ranging from not at all important to their most important motives. Then, we ask participants to judge the extent to which a particular behavior or choice will facilitate or inhibit the achievement of each of the motives. From these judgments we calculate a Goal Impact score, which indicates the extent to which a choice or behavior impacts the participant’s motives.

#### Predicting retirement intentions

Brougham and Walsh [69,70] first demonstrated the power of the Chulef et al taxonomy to predict important life choices. They found that people's Goal Impact scores predicted an additional 20% of the variance in older adults plans for retirement over and above the variance predicted by their age, health, or wealth (accounting for a total of 46% of the variance in retirement intentions). This work demonstrates the robustness of a taxonomy of motives, as similar levels of prediction were achieved with smaller or larger subsets of motives, evaluated by participants in a variety of judgment formats.

#### Changing one’s job

In Talevich, Read, & Walsh [71] we showed that the motive taxonomy, used in conjunction with our Goal Impact measure, could be used to strongly predict people’s intentions to stay in their current job or leave it. That paper presents a summary of our general procedure for using the current taxonomy to predict behavior and it also summarizes results that predict behavior in three additional domains.

#### Health and obesity

Obesity is a major current health problem. Lee, Talevich, Larsen, Lee, Read, and Walsh (in preparation) were able to predict people’s weight from the Goal Impact composite ratings of this taxonomy’s V-level clusters.

#### Coercion and adherence to taking psychotropic medication

Talevich [72] showed that patients better adhered to their medication if doing so was facilitative of their important motives, whereas they were less likely to take their medication when they viewed it as inhibitive of their most important motives.

#### Attachment and close relationships

Attachment theory posits that human bonding is a goal-corrected system that has evolved to procure from caregivers the safety and resources necessary for survival, particularly in the face of a threat. Talevich [73] manipulated threat and perceived responsiveness of an attachment figure. She found that the extent to which the situation (including the attachment figure’s responsiveness) made it harder for them to achieve their motives significantly mediated the relationship between the manipulated situation and attachment behaviors.

#### Limitations

Our empirically generated structure finds many parallels with various theories of human motivation: theories that come from ancient cultures in the east and west, classic psychological theories from the 19th century, as well as those of modern times. That our empirical findings are consistent with so many theories suggests that this structure, though certain to grow and change as we learn more, arises from basic psychological processes shared by human beings throughout the ages and in cultures around the world.

Undoubtedly, there are more motives to be added and, as motives are added, the structure will adjust in kind. That is why we claim no more than that it is *toward* a comprehensive taxonomy of human motives. Our purpose has not been to provide the definitive structure of human motives but a tool for researchers with the greatest breadth of motives, detail, and utility (e.g. zooming in and out at different levels) yet to date.

It would be interesting to administer our similarity judgment task in other cultures. Unfortunately, the complexity and duration (~45–60 minutes) of the similarity judgment task is much, much greater than the importance rating task used by cross-cultural works to date. And using rating scales instead of sorting to measure similarity would not solve the problem. In fact, gathering similarity ratings of all possible motive pairs would be even more expensive and time consuming than using sorting with so many elements. Rating similarity for 161 motives would require 12,880 ratings of all possible pairs of motives ((161*(161–1))/2 = 12,880). Assuming an optimistic 2 seconds a rating, this would take over 7 hours for a complete set of ratings. In contrast, rating the importance of 161 motives only requires 161 ratings and given the same assumption of 2 seconds an item would take less than 6 minutes. Regardless of the actual time per item, the ratio of time required is 80 to 1 for the two tasks.

The ease of scale administration has been a boon to importance-rating based work investigating cultural differences in the allocational properties of motivation ([37–39]). However, our use of similarity judgments was necessary to the creation of a taxonomy based on the structural properties of motivation ([16] see Intro for discussion).

Our purpose was to develop, as much as possible, a context-free structure of motives for researchers to use in the domain of their choosing. Be that domain a cross-cultural analysis of values or understanding why psychiatric patients won’t take their medication, we propose that our taxonomy provides a detailed starting point for selecting the right variables to measure and address many questions about motivated human behavior.

### Conclusion

This taxonomy is a powerful tool for identifying the “right variables” to measure for a limitless range of investigations into human motivation. This starts at the highest level of the taxonomy, which shows that meaning motives are distinct from agency and communion motives—a new finding in the literature.

As we noted in the introduction, the development of a widely accepted taxonomy of traits has been a major focus of personality research and has resulted in major advances in the study of personality. It has greatly aided conceptual organization, theory development, and communication among researchers. Like the Big 5, the current taxonomy should foster the development of the field in several ways. First, it helps provide a common language that should improve communication among researchers. Second, by providing a conceptual structure that identifies how various motives are interrelated, it should help to systematize and integrate this flourishing field of research. Third, attempting to explain this structure should further encourage theory and the development of causal models about the structure. Fourth, the utility of this taxonomy extends beyond the domain of motivation and into personality by examining the relations between the two domains. The Big 5 only addresses traits. It does not address much of what underlies traits: chronic goals and motives (e.g., [12,74]). Thus, our taxonomy could help address central problems such as the relationship between person and situation in behavior. In doing so, it could help integrate two major approaches to studying personality: the trait and the social cognitive [75] approaches.

The current taxonomy has a number of advantages over previous attempts, including our own [45]. It is based on a greater number of motives (161), gathered from a more extensive array of domains (particularly avoidance motives), than has been attempted before (e.g., Cattell’s [31] 16 Ergs; Rokeach’s [32] 36 values). And the structural information provided by our taxonomy is much greater than that provided by other systems (e.g., Murray’s [10] complex list of “variables of personality,” each including distinctly different subvariables). These systems have aimed at covering all areas of human functioning but do not facilitate the analysis of the relationships among the various motives because they provide no information about their hierarchical structure.

Moreover, as a web based study, our taxonomy has a broader sample of participants than previous efforts, which have either relied on undergraduates or smaller non-student samples. Further, it provides a consistent, replicable structure over a range of subjects varying by age and gender. Moreover in contrast to much previous work, it is empirically constructed, rather than being based on the theoretical preconceptions of researchers. This taxonomy provides researchers and practitioners with a broad framework for the study and assessment of human motives, and their role in social behavior.

This taxonomy also enables choice among domains of interest and levels of construal. It offers a common framework from which to sample and reliably measure the human motivators of a wide range of everyday activities. For instance, let us consider the question of how achievement (or lack of achievement) of various motives (e.g. family, career, health, and finances) in different life domains might affect an individual’s satisfaction with those domains. A researcher might measure the extent to which each of these motive clusters is valued by different individuals and then for these individuals measure the extent to which they view their motives as being facilitated or blocked in each life domain (e.g., job, family, romantic, etc.). The taxonomy would also allow us to compare individuals in terms of a common set of motives. Thus, the current taxonomy makes important contributions to a number of different aspects of the study of human behavior.
